# Relationship Between Dietary Protein Source (Soybean Meal vs. Canola Meal) and Meat Quality Traits in Feedlot-Fattened Indigenous Dairy Lambs

**DOI:** 10.3390/vetsci13040327

**Published:** 2026-03-27

**Authors:** Panagiotis Simitzis, Michael Goliomytis, Eirini Tsimpouri, Aphrodite I. Kalogianni, Marianna Lagonikou, Agori Karageorgou, Gregoria Dandoulaki, Efthimios Touranakos, Athanasios I. Gelasakis

**Affiliations:** 1 Laboratory of Animal Breeding and Husbandry, Department of Animal Science, School of Animal Biosciences, Agricultural University of Athens, Iera Odos 75 Str., 11855 Athens, Greece; pansimitzis@aua.gr (P.S.); mgolio@aua.gr (M.G.); akarageorgou@aua.gr (A.K.); gregoriadand@gmail.com (G.D.); efthimist@hotmail.com (E.T.); 2 Laboratory of Anatomy and Physiology of Farm Animals, Department of Animal Science, School of Animal Biosciences, Agricultural University of Athens, Iera Odos 75 Str., 11855 Athens, Greece; etsimpourizootec@gmail.com (E.T.); afrokalo@aua.gr (A.I.K.); marlagonikou@gmail.com (M.L.)

**Keywords:** soybean meal, canola meal, carcass traits, meat quality, oxidative stability

## Abstract

Although soybean meal (SBM) is generally used as the main protein source in livestock diets, canola meal (CM) appears as a sustainable alternative, since it lowers diet cost, especially when regionally produced, while still meeting animal nutritional needs. The objective of this study was therefore to assess the effects of dietary protein source (SBM vs. CM) on carcass traits and meat quality characteristics of feedlot-fattened dairy lambs. A total of 193 weaned lambs, approximately 3 months of age, from two indigenous Greek dairy breeds (75 Chios and 118 Serres), were used. Lambs were randomly assigned to one of two isocaloric and isonitrogenous dietary treatments: a control ration containing SBM as the primary protein source, and an alternative ration in which SBM was completely replaced by CM. After a fattening period of 13 weeks for Chios lambs and 15 weeks for Serres lambs, animals were slaughtered upon reaching a live weight of 35–40 kg, and hot and cold carcass weights were recorded. After 24 h of carcass storage at 4 °C, Longissimus lumborum muscle was sampled and used for the measurement of pH, colour attributes, cooking loss, shear force, and intramuscular fat content. Lipid oxidation was evaluated on days 1, 3, 6, and 9 of refrigerated storage at 4 °C. The substitution of SBM by CM as the main dietary protein source did not affect carcass traits in Serres lambs, whereas CM- treated Chios lambs showed an increased hot and cold carcass weight (*p* < 0.05). Meat quality characteristics were not affected by the dietary treatment in either Chios or Serres lambs, with the exception of meat oxidative stability that was deteriorated in CM compared to SBM Serres lambs (*p* < 0.001). In conclusion, the utilization of canola instead of soybean meal did not negatively influence carcass traits or meat quality characteristics in either Chios or Serres lambs, with the exception of lipid oxidation which was significantly higher in CM supplemented Serres lambs.

## Simple Summary

Soybean meal is widely used as the primary protein source in livestock diets. However, the growing competition between human food needs and livestock feed demands has intensified interest in alternative protein sources that are cost-effective and locally available. This study evaluated the effects of replacing soybean meal with canola meal on carcass characteristics and meat quality parameters in dairy lambs. The findings demonstrate that canola meal is a viable protein source in lamb diets, as its inclusion did not negatively affect carcass traits or meat quality attributes.

## 1. Introduction

Ιn dairy sheep farming systems across the Mediterranean basin, meat production is generally considered a secondary activity to milk production, typically yielding light carcasses of approximately 7–12 kg [1,2]. Nevertheless, the fattening of dairy breed lambs and their slaughter at live weights exceeding 30 kg may offer added value for producers and contribute to meeting the increasing global demand for meat [3]. Consumer preferences for lamb meat are shaped by both intrinsic and extrinsic quality attributes. From the meat industry standpoint, key indicators of meat quality include colour, pH, water-holding capacity, cooking loss, shear force, lipid oxidation, and chemical composition, all of which influence how it behaves during storage and processing and how it is perceived by consumers [4]. A wide range of factors influence both the quantity and quality of lamb meat including breed, age, sex, nutrition, slaughter weight, pre-slaughter handling, slaughter method, and post-slaughter practices such as chilling rate and ageing duration [5]. Among these, nutrition plays a central role, as it directly affects animal performance and meat attributes and represents a major component of production costs in lamb-fattening systems.

In Greece, feedlot fattening, for the production of heavy lambs, is not widely practiced, and existing feedlot operations typically focus on imported specialized meat breeds. Demonstrating that lambs from indigenous dairy breeds can be successfully fattened under feedlot conditions has important implications for the sector, as it provides a valuable opportunity to increase farmers’ income, diversify production strategies, and reduce reliance on imported meat breeds. Moreover, optimizing the fattening process and outcomes in dairy breed lambs could support the development of more sustainable and locally adapted sheep meat production systems utilizing the millions of lambs produced annually. Such systems would capitalize on the genetic resources of indigenous breeds while contributing to the overall resilience and economic viability of the national sheep industry.

Protein-rich feedstuffs such as soybean (SBM) and its by-products dominate livestock diet formulation [6,7]. Member states of the European Union import several million tons of SBM annually, primarily from the USA, due to very low self-sufficiency levels, estimated at roughly 5% [8]. However, rising SBM prices have increased food-feed competition between humans and livestock. Moreover, concerns have been raised regarding its long-term sustainability in animal nutrition due to the environmental impacts associated with soybean cultivation and international trade [9,10]. At the same time, consumers’ scepticism toward the use of genetically modified organisms in animal diets have collectively driven interest in alternative, cost-effective, and locally available protein sources. Canola meal (CM) has emerged as a promising substitute, offering both economic and environmental advantages. CM is generally cheaper than SBM, especially when regionally produced, while still meeting animal nutritional needs. With a crude protein content of approximately 35–40%, CM is considered a high-quality and affordable protein source for livestock diets [11,12,13]. Furthermore, CM utilization is sustainable, since it improves protein efficiency, reduces nitrogen excretion and methane emissions [14], and minimizes dependency on global soy markets.

Despite its potential, relatively few studies have examined the effects of replacing SBM with canola meal (CM) on carcass traits in dairy lambs, and information regarding its influence on meat quality characteristics and oxidative stability remains limited. Therefore, the objective of the present study was to evaluate the effects of substituting SBM by CM as the primary dietary protein source on the meat quality attributes of Greek indigenous dairy-breed lambs.

## 2. Materials and Methods

### 2.1. Animals and Diets

A total of 193 weaned lambs, approximately 3 months old, from two indigenous Greek breeds (75 Chios and 118 Serres) were used as previously described by Tsimpouri et al. [15]. In brief, the study was conducted at a commercial lamb-fattening operation located in Argos, Greece (altitude: 200 m above sea level, latitude: 37°42′37.3″ N and longitude: 22°34′12.9″ E). Lambs were naturally reared for 50–60 days and provided ad libitum access to creep feed and barley straw for 4–8 h daily during morning and early afternoon from the fourth week of age until weaning. Following weaning, a two-week adaptation period was implemented, during which lambs of each breed were allocated in a separate pen. Lambs were then stratified by live weight and sex and randomly assigned to one of the two dietary treatments (groups A and B), resulting in four experimental subgroups: AC and BC (Chios lambs) and AS and BS (Serres lambs) (Table 1). Average initial bodyweights by breed and sex have been previously detailed by Tsimpouri et al. [15]. In brief, bodyweights (±SD) for groups AC, BC, AS, and BS were 21.8 (±3.89), 21.9 (±3.68), 19.1 (±2.76), and 19.1 (±2.72), respectively.

Group A received a concentrate ration containing maize, barley, wheat, wheat bran, and a mineral–vitamin premix with SBM as the main protein source. Group Β received a ration in which SBM was completely replaced by canola meal (CM), and which additionally included citrus pulp, linseed, and carob meal. Both rations were proprietary mixtures (Denezis A.E., Argolida, Greece) and were formulated to be isocaloric based on their metabolizable energy and isonitrogenous, containing 13.2–14.2% crude protein, and 3.1–4.7% crude fat as previously described by Tsimpouri et al. [15] and presented in Table 2. Alfalfa hay and barley straw were provided as forage sources. Concentrates and alfalfa hay were offered twice daily in equal portions and adjusted according to the animals’ nutritional requirements and fattening stage (0.6 to 0.9 kg/day/lamb and 0.3 to 0.4 kg/day/lamb of concentrates and alfalfa hay, respectively, while barley straw was provided ad libitum in both groups.

Following a one-week dietary adaptation period, Chios lambs were fattened for 13 weeks and Serres lambs for 15 weeks, until reaching a target live weight of 35 to 40 kg. Experimental groups were housed in adjacent pens under comparable environmental and management conditions, which were inspected daily to ensure uniformity.

### 2.2. Pre-Slaughter Live Weight and Carcass Traits

Pre-slaughter management procedures, including feed restriction and transportation, as well as slaughtering, are described in detail by Lagonikou et al. [16]. Briefly, lambs were fasted for 12 h with free access to water, weighed, and slaughtered in a local abattoir according to EU animal welfare regulations. Hot carcass weight (HCW) was measured 45 min post-slaughter. After carcass storage for 24 h at 4 °C, cold carcass weight (CCW) was measured and the Longissimus lumborum muscle was excised from the left carcass half for the subsequent analyses. Dressing percentage was calculated as HCW divided by final live weight.

### 2.3. Meat Quality Characteristics

#### 2.3.1. pH 24 and Colour

Muscle pH was measured 24 h post-slaughter using a portable pH metre (HI 99163, Hanna Instruments, Cluj, Romania) calibrated at room temperature using standard buffer solutions at pH 4.0 and 7.0 (Merck, Darmstadt, Germany). The Longissimus lumborum muscle was cut perpendicular to the muscle fibres and allowed to bloom for 30 min at room temperature. Meat colour was then assessed in triplicate using a Miniscan XE chromameter (HunterLab, Reston, VA, USA) operating in the L* (lightness), a* (redness), and b* (yellowness) system (CIE 1976, Commission International de l’ Eclairage). Standard white and black tiles were used for the calibration of the chromameter.

#### 2.3.2. Cooking Loss and Shear Force Value

A sample from the Longissimus lumborum muscle from each lamb (80 ± 2 g) was weighed, sealed in plastic bags, and cooked in a water bath at 75 °C for 35 min. A thermocouple was inserted into the samples, coupled to a digital thermometer model GMH 3710 (Greisinger, Regenstauf, Germany) to monitor the final core internal temperature (70 °C). After this period, samples were cooled under running tap water for 15 min and subsequently equilibrated to room temperature [17]. Each sample was reweighed to calculate the percentage of cooking loss (%). Three subsamples with a cross-sectional area of 1 cm^2^ were then cut parallel to the muscle fibres, and shear force was measured using a Warner-Bratzler (WB) shear blade mounted on a Zwick Testing Machine Model Z2.5/TN1S (Zwick GmbH and Co., Ulm, Germany). Peak shear force values were recorded in Newtons.

#### 2.3.3. Measurement of Intramuscular Fat and Lipid Oxidation—MDA Assay

Intramuscular total lipids were quantified using the method originally described by Folch et al. [18]. Tissue samples were homogenized in a 2:1 *v*/*v* chloroform-methanol solution to achieve a final dilution 20 times the volume of the tissue sample. The crude extract was then mixed with water at 0.2 times its volume and separated into 2 phases. The lower phase contained the extracted tissue lipids and was collected for subsequent analysis.

Lipid oxidation was assessed by measuring malondialdehyde (MDA) concentrations on days 1, 3, 6, and 9 of refrigerated storage at 4 °C (storage time) using a selective third-order derivative spectrophotometric method previously described by Botsoglou et al. [19].

In brief, 2 g from each meat sample (in duplicate) were homogenized (Edmund Buehler 7400 Tuebingen/H04, Bodelshausen, Germany) with 8 mL of aqueous trichloroacetic acid (TCA; 50 g/L) and 5 mL butylated hydroxytoluene (BHT) in hexane (8 g/L). The mixture was centrifuged at 5000× *g* for 5 min, after which the upper hexane layer was discarded. A 2.5 mL aliquot from the bottom layer was mixed with 1.5 mL of aqueous 2-thiobarbituric acid (TBA; 8 g/L) and incubated at 70 °C for 30 min. Following incubation, the samples were cooled under tap water and analyzed using third-order derivative (3D) spectrophotometry (Hitachi U3010 Spectrophotometer, Tokyo, Japan) over the wavelength range of 500–550 nm. Malondialdehyde concentration (ng/g wet tissue) was calculated from the peak height at 521.5 nm using a calibration curve prepared with 1,1,3,3-tetraethoxypropane (TEP).

### 2.4. Statistical Analysis

A mixed model procedure was used to estimate the fixed effects of ration group, sex, and their interaction on final live weight, carcass traits (hot and cold carcass weight and dressing percentage), and meat quality characteristics for each breed, including pH24, colour parameters (L*, α*, b*), IMF, cooking loss, and shear force. The statistical model applied (Model 1) was as follows: *X_ij_* = *μ* + *R_i_* + *S_j_* + *(R × S)_ij_* + *ε_ij,_* where *X_ij_* = the depended variable, μ = overall mean, *R_i_* = fixed effect of ration group *i*, *S_j_* = fixed effect of sex *j*, *(R × S)_ij_* = interaction effect of ration group *i* by sex *j,* and *ε_ij_* = residual error. Malondialdehyde (MDA) concentration was analyzed using a mixed model appropriate for repeated measurements, which included ration group, sex, storage time in the refrigerator, and their interaction as fixed effects. Storage time was the repeated factor, and the animal was included as a random effect. Compound symmetry was selected as the covariance structure based on the Akaike’s Information Criterion. The statistical model applied (Model 2) was as follows: *MDA_ijkl_* = *μ* + *R_i_* + *S_j_* + *T_k_* + *(R × S)_ij_* + *(R × T)_ik_* + *α_l_* + *ε_ijkl_*, where *MDA_ijkl_* = MDA concentration, *μ*= overall mean, *Ri* = fixed effect of ration group i, *S_j_* = fixed effect of sex j, *T_k_* = fixed effect of storage time k, *(R × S)_ij_* = interaction effect of ration group i by sex j, *(R × T)_ik_* = interaction effect of ration group i by storage time k, *α_l_* = random effect of animal l and *ε_ijkl_* = within-animal residual error at time k. All statistical analyses were conducted by SAS/STAT software Version 9.1.3 with statistical significance being set at a = 0.05.

## 3. Results

A positive effect of CM compared to SBM was observed on fattening performance of Chios lambs. This was expressed either as tendency (40.94 vs. 38.87 kg for final live weight and 57.93 vs. 56.84% for dressing percentage, respectively, *p* < 0.1) or as a statistically significant difference (24.28 vs. 22.69 kg for hot carcass weight and 23.68 vs. 22.11 kg for cold carcass weight, respectively, *p* < 0.05; Table 3). In contrast, no significant differences in the examined carcass traits were detected between groups AS and BS, as presented in Table 3.

As indicated in Table S1, a significant effect of sex (*p* < 0.001) was detected in the final live weight of Chios lambs; male lambs exhibited higher final live weight than females. Similar sex-related differences were observed for hot (*p* < 0.001) and cold carcass weight (*p* < 0.001), while dressing percentage was greater in females than males (*p* = 0.001). The interaction between ration group and sex was not significant for any of these traits (*p* > 0.05). A significant sex effect (*p* < 0.001) was also observed in the final live weight of Serres lambs (Table S1); males had higher final live weight than females. Similar effects were shown for hot (*p* < 0.001) and cold carcass weight (*p* < 0.001). On the other hand, dressing percentage was greater in female than male lambs (*p* = 0.005). As with Chios lambs, the ration group × sex interaction was not significant for any parameter (*p* > 0.05).

As shown in Table 4, replacing SBM with CM did not result in significant differences in meat pH, colour attributes (L*-lightness, a*-redness, b*-yellowness), cooking loss, shear force, or IMF content in either Chios or Serres lambs.

In Chios lambs, males exhibited higher shear force values compared with females (Table S2; *p* < 0.001). Sex did not significantly influence pH, L*, a*, b*, cooking loss, or IMF content (Table S2), while no significant interactions of ration group by sex were detected for any trait. Similarly, in Serres lambs, males showed higher shear force values (*p* < 0.01) and L* (*p* < 0.01) compared with females (Table S2). In contrast, IMF content was higher in females than in males (Table S2; *p* = 0.001). Sex was not associated with differences in pH, a*, b*, or cooking loss in Serres lambs, while the interaction of ration group by sex for these parameters was also not significant.

In Chios lambs, MDA values were not significantly affected by the dietary protein source. On the other hand, Serres lambs fed the CM-based diet exhibited higher MDA values on days 3, 6, and 9 of storage (*p* < 0.001, Table 5). Regarding the effects of sex, female Chios lambs showed higher MDA values than males (94.22 ± 4.63 ng/g vs. 81.05 ± 4.37 ng/g; *p* < 0.05). However, no significant sex-related differences were found for MDA in Serres lambs (110.34 ± 6.55 ng/g vs. 123.84 ± 5.79 ng/g). The interaction between ration group and sex was not significant for either breed.

## 4. Discussion

To the best of our knowledge, this study is the first to evaluate the feedlot fattening performance of lambs from two indigenous Greek dairy sheep breeds, Chios and Serres, specifically for the production of heavy carcasses. It also represents the first comprehensive assessment of their carcass traits and meat quality characteristics when fed either a conventional fattening concentrates ration based on SBM as the primary protein source or an alternative one in which SBM was completely replaced by CM.

The replacement of SBM by various grain legumes in fattening lamb diets has been investigated, with most studies reporting no adverse effects on growth performance, carcass traits, or meat quality characteristics [20,21,22,23,24,25]. In agreement with these studies, in our experiment, the complete substitution of SBM with CM did not adversely affect final live weight, carcass traits, or meat quality characteristics in either Chios or Serres lambs. Chios lambs fed with CM tended to exhibit higher final live weight and dressing percentage, while their hot and cold carcass weights were significantly higher than those of lambs fed with SBM. In contrast, no significant differences in final live weight or carcass traits were observed between dietary treatments in Serres lambs. The increased final live weight and carcass yields observed in Chios lambs fed with CM may reflect enhanced fat deposition, improved muscle development or a combination of both compared with SBM-fed lambs. Regardless of the dietary protein source, both breeds produced relatively heavy carcasses, indicating satisfactory fattening performance with potential economic benefits for the dairy sheep sector. These findings align with previously published data showing that live weight tended to increase in group BC compared with group AC after the fourth week of fattening, resulting in higher average daily gain, whereas no significant differences were found between groups AS and BS [15]. Our findings are also consistent with those of Ponnampalam et al. [26], who reported no significant differences in final live weight or hot carcass weight in crossbred lambs ([Merino × Border Leicester] × Poll Dorset) fed diets supplemented with either CM or SBM and slaughtered at comparable weights with our experiment (36–40 kg).

As previously reported, the inclusion of full-fat canola at levels up to 18% in the diets of finishing South African Mutton Merino lambs did not negatively affect average daily gain (ADG) or feed conversion ratio (FCR), indicating that canola can serve as an excellent source of both protein and energy [27]. These findings are also supported by Stanford et al. [28], who observed that replacing soybean meal with canola as the primary protein source had no significant effect on FCR, while ADG increased in Romanov x Suffolk lambs. Other studies have reported improved feed efficiency of fattening lambs when canola meal was included in the diets at 12% [29] or 28% [30], suggesting that canola may enhance the anabolic processes in fattening lambs. On the contrary, in a more recent study, it was found that inclusion of canola at the level of 16% may negatively affect hot and cold carcass yields, leading to the recommendation that canola meal should be added at no more than 8% in diets for Santa Ines lambs to avoid adverse effects on performance and carcass traits [31].

According to Sekali et al. [32], partial or complete replacement of SBM with heat-treated canola meal in the diets of Meatmaster lambs did not negatively affect growth performance, carcass traits (hot and cold carcass weight and dressing percentage), or meat quality characteristics (pH, colour, water holding capacity, cooking loss, and tenderness). However, the same authors in a previous study reported that partial replacement of SBM with CM in Mutton Merino lambs improved hot and cold carcass weights, while final live weight, meat pH, and colour parameters remained unaffected [33]. Similarly, dos Santos Penha et al. [34] found that total substitution of SBM by canola grain did not affect meat pH, cooking loss, shear force, or colour attributes (lightness, redness, and yellowness), which is in accordance with our findings. Moreover, replacing lupins with CM in Poll Dorset x Texel lamb diets did not have significant effects on dressing percentage, muscle pH, or meat colour [29].

Although no significant differences in meat quality characteristics were observed between lambs fed with SBM or CM in our study, pH values were within the acceptable range of 5.5–5.8 [35]. Likewise, lightness values were within the acceptable range for fresh lamb meat (≥34; [36]), and redness values exceeded the threshold of ≥9.5 for consumer acceptability [36].

Moreover, MDA values were significantly higher only in Serres lambs fed with CM compared with those fed with SBM on days 3, 6, and 9 of storage. This increase may be attributed to the higher proportion of polyunsaturated fatty acids (PUFAs), that are more susceptible to oxidation, in the canola meal-based diet due to the inclusion of linseed. Linseed is rich in alpha-linolenic and linoleic acids (approximately 56 and 16%, respectively) [37], and its dietary supplementation has been associated with increased meat TBARS values in lambs [38]. Despite the increased MDA values observed in lambs fed with CM, all values remained below the acceptable threshold of 2.5 mg /kg meat suggested for beef.

Breed-related differences in growth performance, carcass traits, and meat quality characteristics have previously been reported between fat- and thin-tailed Greek indigenous breeds [39]. However, the design of the present study does not permit direct comparisons between the two breeds as the fattening periods differed; consequently, breed and time effects are confounded and cannot be evaluated independently. A larger-scale study including lambs from both breeds at the same fattening period and operation is required to examine possible differences between the breeds.

Regarding the effect of sex, male lambs exhibited significantly higher final live weight as well as greater hot and cold carcass weights compared to females in both breeds, likely associated with their superior feed efficiency and faster growth rate [40,41]. However, sex did not have a significant effect on meat pH, colour attributes (redness and yellowness), or cooking loss. Similarly, Horcada et al. [42] reported no significant differences in meat pH, colour parameters, or water holding capacity between male and female Lacha and Rasa Aragonesa lambs. In another study involving Bragancana and Mirandesa lambs, meat pH, redness, and yellowness were also comparable between sexes. Consistent with our results, several studies have found that shear force values are higher in male than in female lambs [43,44,45,46]. On the other hand, IMF content was lower in male compared to female Serres lambs, more likely associated with the precocity of adipose tissue deposition in females. This pattern is further supported by evidence that the expression of key lipogenic genes, such as lipoprotein lipase (LPL), acetyl-CoA carboxylase alpha (ACACA), fatty acid synthase (FASN), and stearoyl-CoA desaturase (SCD) is elevated in female compared to male lambs [47]. Sex-related differences in IMF of Longissimus dorsi muscle have been reported in several studies, despite variations in anatomical regions and slaughter weights [42,45,46,48,49,50]. According to Hopkins et al. [51], IMF is negatively correlated with shear force, which supports the pattern observed in our study.

In Serres lambs, meat lightness was higher in males compared to females, which is consistent with previous findings [52,53,54]. Lightness depends on pigment components, such as hematin, myoglobin, and their chemical forms. Chromophores, especially myoglobin and hemoglobin, absorb visible light, leading to reduced reflectance [55]. Lower myoglobin concentrations and consequently higher lightness values have been found in meat from male lambs compared with females, indicating a clear sex effect on meat lightness [56].

Moreover, MDA values were higher in female than in male lambs, although the difference reached significance only in Chios lambs. The elevated MDA values observed in female lambs of both breeds may be related to their higher IMF content. Lipid oxidation is influenced by several factors, including IMF percentage, the fatty acid composition of Longissimus lumborum muscle, pro-oxidant levels, and breed, all of which should be considered when evaluating oxidative stability [57].

## 5. Conclusions

In conclusion, the replacement of CM by SBM did not negatively affect carcass traits or meat quality characteristics in either Chios or Serres lambs, with the exception of lipid oxidation which was significantly higher in CM supplemented Serres lambs. As indicated, CM can generally serve as a viable alternative protein source in lamb fattening diets, with potential benefits for both economic efficiency and environmental sustainability. Future research is warranted to elucidate the effects of replacing SBM with CM across different slaughter ages and weights, particularly in relation to fattening performance, meat quality, and overall financial sustainability.

## Figures and Tables

**Table 1:** Number of Chios and Serres lambs per ration group and sex.

	Ration Groups *			
	AC	BC	AS	BS
Males	24	16	33	34
Females	15	20	25	26
Total	39	36	58	60

* AC: Soybean meal—Chios; BC: Canola meal—Chios; AS: Soybean meal—Serres; BS: Canola meal—Serres.

**Table 2:** Chemical composition of the rations fed in groups A (ration with soybean meal) and B (ration with canola meal) in the two studies [15].

	Ration A	Ration B
Chios lambs		
Dry matter (%)	91.9	90.6
Ash (%DM)	5.7	5.4
Crude protein (%DM)	14.2	13.7
Fat (%DM)	3.1	4.2
Crude fibre (%DM)	3.3	4.7
Serres lambs		
Dry matter (%)	91.7	91.1
Ash (%DM)	5.6	8.2
Crude protein (%DM)	13.2	13.3
Fat (%DM)	3.3	4.7
Crude fibre (%DM)	3.5	6.1

**Table 3:** LS Means ± SEM * of final live weight and carcass traits in Chios and Serres lambs fed rations with different dietary protein sources (soybean vs. canola meal).

Parameter	Diet		SEM	*p*-Value
	Soybean Meal—Based Ration	Canola Meal—Based Ration		
Chios lambs				
Final live weight (kg)	38.87	40.94	0.82	0.077
Hot carcass weight (kg)	22.69	24.28	0.52	<0.05
Cold carcass weight (kg)	22.11	23.68	0.51	<0.05
Dressing Percentage (%)	56.84	57.93	0.40	0.055
Serres lambs				
Final live weight (kg)	36.82	36.85	0.57	NS **
Hot carcass weight (kg)	21.77	21.47	0.35	NS
Cold carcass weight(kg)	21.17	20.94	0.34	NS
Dressing Percentage (%)	57.68	56.80	0.43	NS

* Least Square Means ± standard error of the means ** NS: Non-significant.

**Table 4:** LS Means ± SEM * of meat quality characteristics in Chios and Serres lambs fed rations with different dietary protein sources (soybean vs. canola meal).

Parameter	Diet		SEM	*p*-Value
	Soybean Meal—Based Ration	Canola Meal—Based Ration		
Chios lambs				
pH	5.72	5.74	0.01	NS **
L* (lightness)	38.97	39.24	0.47	NS
a* (redness)	11.33	11.34	0.20	NS
b* (yellowness)	13.00	12.88	0.14	NS
Cooking loss (%)	16.87	15.62	0.40	NS
Shear force value (Ν)	23.69	22.88	1.30	NS
Intramuscular fat content (%)	3.63	3.77	0.20	NS
Serres lambs				
pH	5.70	5.69	0.01	NS
L* (lightness)	38.28	38.31	0.39	NS
a* (redness)	11.76	11.76	0.15	NS
b* (yellowness)	12.66	13.08	0.16	NS
Cooking loss (%)	21.08	21.61	0.51	NS
Shear force value (Ν)	46.72	45.86	1.05	NS
Intramuscular fat content (%)	2.94	3.06	0.13	NS

* Least Square Means ± standard error of the means; ** NS: Non-significant.

**Table 5:** LS Means ± SEM * of meat oxidative stability (ng MDA/g meat) in Chios and Serres lambs fed rations with different dietary protein sources (soybean vs. canola meal).

Storage at 4 °C	Diet		SEM	*p*-Value
	Soybean Meal—Based Ration	Canola Meal—Based Ration		
Chios lambs				
Day 1	24.55	20.27	5.53	NS **
Day 3	77.56	87.40	5.53	NS
Day 6	112.45	118.46	5.53	NS
Day 9	126.99	133.41	5.53	NS
Days 1–9	86.99	88.28	4.54	NS
Serres lambs				
Day 1	3.53	5.96	8.50	NS
Day 3	62.29	92.70	8.50	<0.001
Day 6	114.34	165.78	8.50	<0.001
Day 9	195.94	296.19	8.50	<0.001
Days 1–9	94.03	140.16	6.21	<0.001

* Least Square Means ± standard error of the means; ** NS: Non-significant.

## Data Availability

The original contributions presented in this study are included in the article/Supplementary Materials. Further inquiries can be directed to the corresponding author.

## Supplementary Materials

The following supporting information can be downloaded at https://www.mdpi.com/article/10.3390/vetsci13040327/s1, Table S1: LS Means ± SEM* of final live weight and carcass traits in male and female Chios and Serres lambs.. Table S2: LS Means ± SEM* of meat quality characteristics in male and female Chios and Serres lambs
