# Supplementary material for: Relationship Between Dietary Protein Source (Soybean Meal vs. Canola Meal) and Meat Quality Traits in Feedlot-Fattened Indigenous Dairy Lambs
Source: Vet Sci. 2026 Mar 27;13(4):327. doi: 10.3390/vetsci13040327 (PMC13119829; doi:10.3390/vetsci13040327)
Supplement: Supplementary file 1 [file vetsci-13-00327-s001.zip › vetsci-4119067-supplementary.pdf]

veterinary sciences

Supplementary Materials

Relationship between dietary protein source (soybean meal vs. canola meal) and meat quality traits in feedlot-fattened indigenous dairy lambs

Panagiotis Simitzis[1], Michael Goliomytis[1], Eirini Tsimpouri[2], Afroditi I. Kalogianni[2], Marianna Lagonikou[2], Agori Karageorgou[1], Gregoria Dandoulaki[1], Efthimios Touranakos[1] and Athanasios I. Gelasakis[2,*]

[1] Laboratory of Animal Breeding and Husbandry, Department of Animal Science, School of Animal Biosciences, Agricultural University of Athens, Iera Odos 75 Str., 11855 Athens, Greece; pansimitzis@aua.gr (P.S.); mgolio@aua.gr (M.G.); akarageorgou@aua.gr (A.K.); gregoriadand@gmail.com (G.D.); efthimist@hotmail.com (E.T.[1])

[2] Laboratory of Anatomy and Physiology of Farm Animals, Department of Animal Science, School of Animal Biosciences, Agricultural University of Athens, Iera Odos 75 Str., 11855 Athens; etsimpourizootec@gmail.com (E.T.[2]); afrokalo@aua.gr (A.I.K.); marlagonikou@gmail.com (M.L)

* Correspondence: gelasakis@aua.gr (A.G.); Tel.: +30-21-0529-4387

Table S1. LS Means ± SEM* of final live weight and carcass traits in male and female Chios and Serres lambs.

Parameter	Sex		SEM	P-value
	Male	Female		
Chios lambs				
Final live weight (kg)	42.97	36.84	0.81	<0.001
Hot carcass weight (kg)	24.88	22.09	0.51	<0.001
Cold carcass weight (kg)	24.25	21.54	0.51	<0.001
Dressing Percentage (%)	56.46	58.31	0.39	0.001
Serres lambs				
Final live weight (kg)	39.26	34.41	0.56	<0.001
Hot carcass weight (kg)	22.69	20.55	0.35	<0.001
Cold carcass weight(kg)	22.12	19.99	0.34	<0.001
Dressing Percentage (%)	56.37	58.11	0.43	0.005

*Least Square Means ± standard error of the means

Academic Editor: Firstname Lastname

Received: date
Revised: date
Accepted: date
Published: date

Copyright: © 2026 by the authors. Submitted for possible open access publication under the terms and conditions of the Creative Commons Attribution (CC BY) license.

Table S2. LSMeans ± SEM* of meat quality characteristics in male and female Chios and Serres lambs

Parameter	Sex		SEM	P-value
	Male	Female		
Chios lambs				
pH	5.73	5.72	0.01	NS**
L* (lightness)	39.56	38.65	0.47	NS
a* (redness)	11.25	11.42	0.20	NS
b* (yellowness)	12.91	12.96	0.14	NS
Cooking loss (%)	16.67	15.82	0.38	NS
Shear force value (N)	27.35	19.49	1.27	<0.001
Intramuscular fat content (%)	3.44	3.97	0.20	NS
Serres lambs				
pH	5.71	5.70	0.01	NS
L* (lightness)	39.02	37.57	0.38	<0.01
a* (redness)	11.62	11.90	0.15	NS
b* (yellowness)	12.73	13.00	0.16	NS
Cooking loss (%)	21.29	21.40	0.51	NS
Shear force value (N)	48.24	44.31	1.06	<0.01
Intramuscular fat content (%)	2.68	3.32	0.13	0.001

*Least Square Means ± standard error of the means **NS: No significant

Disclaimer/Publisher's Note: The statements, opinions and data contained in all publications are solely those of the individual author(s) and contributor(s) and not of MDPI and/or the editor(s). MDPI and/or the editor(s) disclaim responsibility for any injury to people or property resulting from any ideas, methods, instructions or products referred to in the content.
